# Variations in services and intervention pathways for traumatic stress in Welsh prisons: A national survey

**DOI:** 10.1177/00258172231214432

**Published:** 2024-02-09

**Authors:** Natasha Kalebic, Clare Crole-Rees, Jack Tomlin, Claudia Berrington, Isidora Popovic, Andrew Forrester

**Affiliations:** 1 Department of Psychological Medicine and Clinical Neurosciences, Cardiff University School of Medicine, Cardiff, UK; 2 School of Law and Criminology, University of Greenwich, London, UK

**Keywords:** Prisons, PTSD, C-PTSD, trauma, criminal justice, clinical pathways, trauma-informed, staff views, mental health

## Abstract

Both Post-Traumatic Stress Disorder and Complex Post-Traumatic Stress Disorder are prevalent in prison settings. Both often go undetected and untreated, while prisoners who already suffered previous trauma may be re-traumatised upon imprisonment. The current study aimed to conduct a national survey of all Welsh prisons to gather information about existing services and treatments for traumatic stress. The survey identified variation within Welsh prisons with regard to NICE-recommended evidence-based therapies. It is therefore recommended that there needs to be development of a pathway of Post-Traumatic Stress Disorder and Complex Post-Traumatic Stress Disorder in the prison system which should be achieved through a consensus process of both frontline staff and experts in the field.

## Introduction

Post-Traumatic Stress Disorder (PTSD) is a complex psychiatric disorder which, according to the International Classification of Diseases, Eleventh Revision (ICD-11), may develop following exposure to an extremely threatening or horrific event or series of events. Additionally Complex Post-Traumatic Stress Disorder (C-PTSD) now exists as a categorical diagnosis in the ICD-11,^1^ and is more likely to develop if the trauma has been an ongoing event, or series of traumatic events, with a recent study in a large medium-secure prison finding that C-PTSD is more frequent among male prisoners (16.7%) than diagnoses of non-complex PTSD (6.7%).^2^ This compares to worldwide rates of 0.5–7.7% for C-PTSD and 3.0–4.4% for PTSD in the general population.^3^

Both PTSD and C-PTSD, while being more prevalent in a prison setting, are often under-identified and therefore untreated.^2^ So, while something about the detrimental effect of trauma and PTSD/C-PTSD on prisoners is known, the exact provisions prisons are able to provide in the identification and treatment of traumatic stress and PTSD/C-PTSD are not.

Therefore, the current study aimed to conduct a national survey of all Welsh prisons to gather information about existing services and treatments for traumatic stress, and discover gaps within trauma-informed practice, service delivery and workforce training to help understand exactly what is currently in place before undertaking further research and making pathways recommendations.

## Methods

### Ethical approval

The project was undertaken as a service evaluation, endorsed by Cwm Taf Morgannwg University Health Board's Research and Development Department (CT/1582/22).

### Design and recruitment

An electronic descriptive survey was developed via Microsoft Forms with quantitative and qualitative responses (56 questions) and emailed to service managers and senior clinicians within each Welsh prison (N = 6) in February 2022.

### Survey

The survey consisted of four main sections: closed tick boxes or free text, adapted from a survey methodology described by McKenna et al.^4^

- **Section 1:** general background information, describing the prison establishment and main mental health providers.
- **Section 2:** details of the service structure.
- **Section 3:** details of staffing levels within each prison's mental health care department.
- **Section 4:** types of mental health interventions available.

### Data analysis

Counts from the Excel sheet of responses were used to describe quantitative data. Qualitative data responses were too few to conduct a rigorous thematic analysis. Therefore, a simple summary of respondents' answers is provided, grouped according to whether barriers or opportunities to implementing trauma-informed practices were described.

## Results

Table 1 shows the category and capacity of each Welsh prison. All prisons in Wales are male-only prisons; female prisoners are housed in England.

**Table 1.** Category and operational capacity of Welsh prisons.^10^

Prison name	Category	Operational capacity
HMP Prescoed	D	260^a^
HMP Usk	C	254^a^
HMP Swansea	B/C	499^b^
HMP Cardiff	B	779^c^
HMP Parc	B	1639^d^
HMP Berwyn	C	1865^d^

a Prison Inspectorate report 2021.

b Prison Inspectorate Report 2020.

c Prison Inspectorate Report 2019

d Prison Inspectorate report 2022.

### Service structure

All responders stated that their respective prisons have a primary and secondary structure to their mental health service, with most integrating primary and secondary care (n = 4). Multi-agency reviews for complex cases were common (n = 5). Nearly all prisons had centrally administered systems in place for the multi-disciplinary or multi-agency management of self-harm and suicide risk (n = 5). Of those, the majority were led by either the prison service (n = 2) or both the health and prison service combined (n = 2). Only one prison stated that they had an inpatient hospital wing that housed mental health patients with a capacity of 20. This inpatient service is configured as “all cells”, and the percentage of inpatients (at the time of response) there primarily for a mental health problem was 60%.

### Staffing

All prisons indicated that nursing and psychiatry were included in their specialist mental health/in-reach model or integrated primary and secondary model, with other professional groups included being psychology (n = 5), occupational therapy (n =3) and clinical pharmacy (n = 2).

### Types of mental health interventions available

Five of the prisons have access to psychological treatments or talking therapies such as counselling, cognitive behavioural therapy (CBT) and eye movement desensitisation and reprocessing (EMDR), and provide psychoeducation programmes for prisoners with mental health problems, which involve:

“the provision of information and advice about a disorder and its treatment. It usually involves an explanatory model of the symptoms and advice on how to cope with or overcome the difficulties a person may experience. It is usually of brief duration, instigated by a healthcare professional, and supported by the use of written materials.”^5^

Three of the prisons stated that they had a screening process for the identification and detection of PTSD/C-PTSD and the identification of traumatic experiences and asked about the experience of trauma as part of the screening process. It is worth noting here that one prison responded with “unknown”.

All prisons provided specific interventions for people with PTSD or C-PTSD. Interventions included guided self-help for PTSD, emotional stabilisation, mentalisation-based therapy, trauma-focused CBT, and EMDR.

All prisons prescribe medication for the treatment of PTSD and psychiatrists were always listed as prescribers. In two prisons, general practitioners (GPs) were also listed as prescribers.

Half of the respondents stated that they use a trauma/psychologically-informed approach within their establishment (n = 3) and those who responded “yes” also stated that their health service uses a trauma-informed/psychologically-informed approach. One prison did state “not known” for both statements so we cannot say for sure that half of the prisons do not. Additionally, the same respondents stated that staff have reflective practice sessions/supervision where they consider the impact of trauma.

### Qualitative responses

Improvements and barriers were identified in the free-text question responses. Thirteen comments were submitted from six respondents across three questions. Quotes are reported to give a full accounting of the survey, but the small sample means a lack of generalisable insights.

Staffing, resources, and organisation were identified as common barriers. Respondents noted that “prison staff [lacked] understanding of interventions available”. Additionally, with low funds, “training in EMDR [is] expensive” and prisons need to “target resources”. Shorter prison sentences were also identified as a barrier with “issues relating to the fact the prison is a remand one, so time is of the essence”.

Staffing, prevention, trauma-informed practice, identification, and intervention were recognised as possible areas for improvement. Staff training was mentioned six times. For example, respondents stated hopes of “maintaining staff who are trained in appropriate interventions, not just the identification of symptoms”. Respondents wanted specific measures to reduce “action of self-harm and violence” due to residents experiencing “trauma in childhood”. They also identified a need for “more psychological therapy in primary care” and the “assessment [and] identification of need signpost[ed] into [the] appropriately trained staff to deliver interventions”. Improvements in identification and intervention were proposed twice, with one respondent noting “early identification and intervention [being] combined” and, more specifically, advances in “identifying and treating past trauma”. Finally, one respondent explained that they are “currently working with Traumatic [Stress] Wales to look at how [they] can introduce a trauma-informed model for the prison”.

## Discussion

PTSD and C-PTSD are common in prison settings,^2,6^ and often go undetected^7^ and untreated.^8^ Only three of the prisons in the current survey stated definitively that they had a screening process for the identification of traumatic experiences as well as identifying and detecting PTSD/C-PTSD and asking about experiences of trauma as part of the screening process. Implementing a standardised method of identification, which recognises the complexities and co-morbidities of PTSD and C-PTSD, could improve detection and treatment.

Variation within Welsh prisons appears to exist in accessing the NICE-recommended evidence-based therapies which are recommended to effectively treat PTSD and C-PTSD. NICE guidance (2018)^9^ recommends the use of trauma-focused psychological treatments for PTSD in adults, specifically the use of EMDR and trauma-focused cognitive behavioural therapy (CBT). Further, short sentences in remand prisons have the disadvantage of causing difficulty in completing trauma therapy when it is offered. Again, there should be implementation of standardised guidelines regarding the interventions made available to those in prison with a history of trauma or a diagnosis of PTSD/C-PTSD.

## Strengths and limitations

Although the relatively small number of prisons could be considered a limitation, this survey covers all prisons in Wales so is a whole nation approach which could be extended out easily. McKenna et al,^4^ on which this study was based, surveyed 19 prisons throughout New Zealand, so rolling this research out across the UK in the first instance could give a broader assessment.

## Future aims and key recommendations

There are evidently gaps in treatment and management of PTSD and C-PTSD within the prison system and we need to understand where and when treatment should be given. Further research is necessary to help identify the current boundaries and restrictions of mental health in-reach service structure as regards the identification and management of PTSD and C-PTSD within prisons, to help create a better equipped national model to support prisoners coping with their trauma exposure and facilitate rehabilitation and help us to understand when intervention is indicated, and when it is not. This can be helped by development of services and training of all frontline staff. There needs to also be development of a pathway of PTSD and C-PTSD in the prison system which should be achieved through a consensus process of both frontline staff and experts in the field.

## Data Availability

The data that support the findings of this study are available on request from the corresponding author. The data are not publicly available due to privacy or ethical restrictions.
